# Multi-Scale Study on Ultrasonic Cutting of Nomex Honeycomb Composites of Disc Cutters

**DOI:** 10.3390/ma18153476

**Published:** 2025-07-24

**Authors:** Yiying Liang, Feng Feng, Wenjun Cao, Ge Song, Xinman Yuan, Jie Xu, Qizhong Yue, Si Pan, Enlai Jiang, Yuan Ma, Pingfa Feng

**Affiliations:** 1 Division of Intelligent Instrument and Equipment, Shenzhen International Graduate School, Tsinghua University, Shenzhen 518055, China; liangyy23@mails.tsinghua.edu.cn (Y.L.); xu-j22@mails.tsinghua.edu.cn (J.X.); leqz24@mails.tsinghua.edu.cn (Q.Y.); ps24@mails.tsinghua.edu.cn (S.P.); jel20@mails.tsinghua.edu.cn (E.J.); 2 AVIC Chengdu Aircraft Industrial (Group) Co., Ltd., Chengdu 610073, China; 13678026768@163.com (W.C.); rommel_sg@163.com (G.S.); yz880417@163.com (X.Y.); 3 Shenzhen Tsingding Technology Co., Ltd., Shenzhen 518133, China; ma.yuan@tsingding.com

**Keywords:** Nomex honeycomb composites, ultrasonic cutting, disc cutter, cutting mechanism

## Abstract

To address the issues of burr formation, structural deformation, and tearing in the conventional machining of Nomex honeycomb composites, this study aims to clarify the mechanisms by which ultrasonic vibration-assisted cutting enhances machining quality. A multi-scale analysis framework is developed to examine the effects of ultrasonic vibration on fiber distribution, cell-level shear response, and the overall cutting mechanics. At the microscale, analyses show that ultrasonic vibration mitigates stress concentrations, thereby shortening fiber length. At the mesoscale, elastic buckling and plastic yielding models show that ultrasonic vibration lowers shear strength and modifies the deformation. A macro-scale comparison of cutting behavior with and without ultrasonic vibration was conducted. The results indicate that the intermittent contact effect induced by vibration significantly reduces cutting force. Specifically, at an amplitude of 40 μm, the cutting force decreased by approximately 29.7% compared to the condition without ultrasonic vibration, with an average prediction error below 8.6%. Compared to conventional machining, which causes the honeycomb angle to deform to approximately 130°, ultrasonic vibration preserves the original 120° geometry and reduces burr length by 36%. These results demonstrate that ultrasonic vibration effectively reduces damage through multi-scale interactions, offering theoretical guidance for high-precision machining of fiber-reinforced composites.

## 1. Introduction

Nomex honeycomb composites (NHCs) are fabricated by blending aramid short fibers with aramid pulp in a specific ratio. The disordered orientation of the internal fibers significantly enhances the strength and toughness of the material. In addition, the unique thin-walled honeycomb structure, through its tight integration with the upper and lower skins, significantly reduces the overall weight while making the material with excellent high specific strength, stiffness, impact resistance, and fatigue resistance [1,2,3,4,5,6]. Based on these excellent characteristics, NHCs have shown significant application value in aerospace key components such as frames, blades, and wings that require high precision machining and adaptability to complex profiles [7,8,9,10]. However, NHCs are considered difficult-to-machine materials due to their thin-walled, cellular structure and low in-plane stiffness along the hexagonal cell direction. When using conventional high-speed milling, there are common processing defects such as fiber tearing and edge burrs, which seriously affect the surface integrity of the component [11,12,13] and reduce strength by 30% [14]. In recent decades, ultrasonic vibration-assisted (UVA) cutting has become a cutting-edge solution for improving the honeycomb processing performance of aramid paper due to its advantages of effectively reducing cutting force, improving machining surface quality, and reducing dust pollution [15,16,17,18,19,20,21]. Among various tool types, disc cutters (DC) are commonly used for surface machining [22,23,24,25,26,27]. Despite these promising developments, the underlying mechanisms by which ultrasonic vibration (UV) influences cutting behavior across multiple scales remain insufficiently understood. Specifically, the effects of ultrasonic excitation on fiber-level morphology, cell-level deformation, and macro-level force responses have not been systematically analyzed. Additionally, the lack of a comprehensive theoretical framework to qualitatively analyze these multi-scale effects limits the optimization and broader application of UVA strategies for NHCs.

To address these challenges, recent studies have established different scale models to investigate the influence of ultrasonic vibration on the cutting mechanics of NHCs. On a macroscopic scale, Xiang et al. [28] found that ultrasonic longitudinal torsional composite vibration can more effectively reduce cutting forces and improve surface quality compared to single longitudinal vibration or traditional cutting methods. Among them, the slip effect plays a positive role in the process of cutting fibers, which can reduce the occurrence of burrs and tear defects. Wang et al. [29] further pointed out that the inclination angle of the disc cutter has a significant impact on the machining residual height and hole wall tearing situation, and reasonable control of the spatial orientation of the cutter is an important means to achieve high-quality machining. In addition, Zarrouk, T. et al. [30,31,32,33] conducted a comprehensive study on the effects of ultrasonic vibration amplitude on stress distribution, chip formation, surface integrity, and cutting force components. Ahmad et al. [34] clarified the influence of parameters such as feed rate, cutting depth, and amplitude on cutting force and surface quality through orthogonal experiments and multi-factor analysis methods. The analysis of cutting force at the macroscopic scale not only reveals the changes in energy consumption and material response during the machining process but also provides the theoretical basis and experimental support for subsequent multi-scale modeling and process optimization. At the mesoscopic scale, Xu et al. [35] explored the transverse and longitudinal cracks introduced by mesoscale mechanical processing, with a particular focus on their formation mechanisms and evolution. Compared to macroscopic approaches, mesoscale models offer more detailed insights into mechanical behavior. As Roy et al. [36] pointed out, accurately capturing honeycomb buckling in jointed structures is critical for design, and representing the honeycomb geometry at the mesoscale using shell elements in finite element models significantly improves simulation accuracy. At the microscopic scale, Qin et al. [37] quantitatively addressed microscopic burr interference by proposing a dimensionality reduction and regression-based method to accurately extract cell contours. Building on this, Dong et al. [38] developed a corner-type-based recognition algorithm, further enhancing structural identification accuracy and robustness against burr-induced distortions.

Although previous studies have explored the process response and defect formation mechanism of UVA cutting of NHCs from different perspectives such as macroscopic cutting experiments, mesoscopic models, and microscopic observations, most of the work has focused on the influence of single scale or specific parameters, lacking a systematic analysis of the synergistic mechanism of ultrasonic vibration in multi-scale structures. Therefore, it is urgent that we establish a unified multi-scale analysis framework to deeply reveal the effects of ultrasonic vibration on processing performance from the perspectives of microscopic burrs formation, mesoscopic structural deformation, and macroscopic mechanical response.

In this work, a multi-scale analysis is established to investigate the cutting mechanisms of UVA machining of NHCs. In Section 2, a multi-scale framework is proposed to qualitatively analyze the influence of ultrasonic vibration, integrating fiber-scale distribution, meso-scale shear deformation, and macro-scale cutting responses. Section 3 presents the materials and methods, where an ultrasonic vibration-assisted cutting platform is constructed, key parameters such as feed rate, spindle speed, and amplitude are defined, and comparative experiments are systematically conducted. In Section 4, experimental results are interpreted using the multi-scale model to reveal changes in cutting forces, cell wall damage, and burr length. A data-driven empirical equation is then formulated and validated by varying parameters and comparing the predicted trends with experimental observations.

## 2. Multi-Scale Analysis

**Figure 1:**
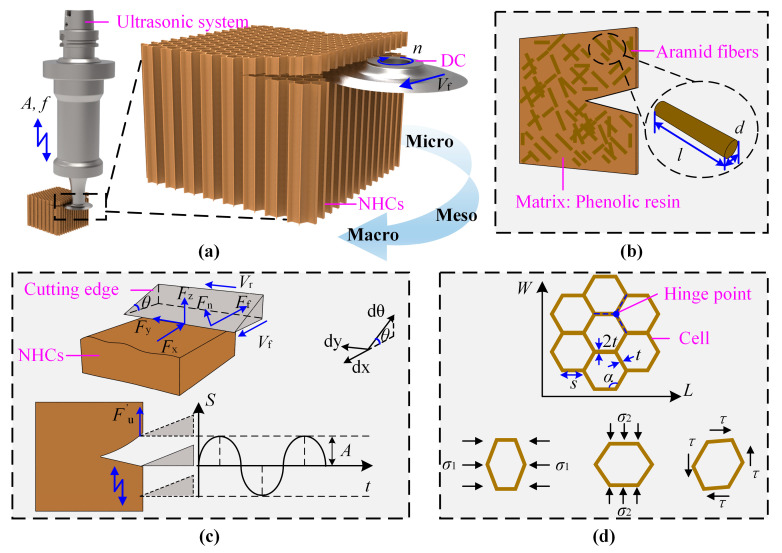
Multi-scale analysis of NHCs with disc cutter: (**a**) schematic of the UVA cutting process; (**b**) microscopic scale shows fiber-matrix morphology; (**c**) macroscopic scale shows mechanical analysis and intermittent cutting behavior under ultrasonic vibration; and (**d**) mesoscopic scale shows deformation of honeycomb cells.

Ultrasonic vibration-assisted cutting typically operates at a high frequency of around 20 kHz, where vertical vibrations are applied to the disc cutter, as illustrated in Figure 1a. To qualitatively analyze the role of ultrasonic vibration during the machining of Nomex honeycomb composites, it is necessary to understand the material response across multiple structural scales. At the microscopic scale, the distribution of fibers and resin matrix affects local stress concentrations, which, in turn, influence burr initiation and removal. At the mesoscopic scale, the geometry and deformation behavior of individual honeycomb cells govern the local structural response. At the macroscopic scale, ultrasonic vibration may affect the overall cutting force and surface quality. Although the mechanisms at each scale differ, they are interrelated to some extent. Ultrasonic vibration may intervene through multiple pathways and contribute to key processes such as burr formation, structural deformation, and material removal. Therefore, a multi-scale analysis framework is established in this study to qualitatively explore the potential effects of ultrasonic vibration at each scale and to reveal its multi-level influence on material behavior.

### 2.1. Microscopic Scale: Fiber Distribution and Matrix Interaction

The microscopic scale concerns the material’s internal composition, including fiber orientation, length, and bonding with the matrix. The NHCs are mainly composed of irregularly arranged short-cut aramid fibers and a resin matrix, and their mechanical behavior is highly dependent on the spatial distribution characteristics of the fibers and the interfacial cooperative behavior. As shown in Figure 1b, due to the non-directional and non-periodic distribution of fibers inside the material, they form complex stress transmission paths under external loads, further affecting the evolution process of micro damage. Especially during the processing, the micro damage mechanisms such as interface debonding, matrix delamination, and fiber pull-out are closely related to the local density of fiber distribution. The *K* function (second-order strength function) [39,40] can describe the aggregation or dispersion characteristics of fiber distribution, and the expression of *K*(*h*) is:

(1) $$K\left(h\right)=\frac{A}{N^{2}}\sum_{i=1}^{N}w_{i}^{-1}L_{i}\left(h\right)$$

where *A* is the area of the observation area, *N* is the total number of fibers in the observation area, and *L_i_*(*h*) is the number of fibers within a given radial distance *h*. Because the boundary effect of the actual observation window can cause bias in the neighborhood statistics of fibers at the edge, *w*_i_ is the boundary correction coefficient, defined as the ratio of the circumference contained within the window to the total circumference of the circle.

For the Completely Spatial Random (CSR) distribution pattern of aramid fibers, the above equation can be simplified as:

(2) $$K_{CSR}(h)=\pi h^{2}$$

Due to the resin coating and solidification of fibers in NHCs, the *K* function is used to describe the relative distribution trend between different processing conditions or damaged areas, providing a basis for the subsequent construction of functions related to distribution characteristics. Furthermore, the *K* function characterizes the local density distribution of fibers, which significantly affects the uniformity of the local stress. Therefore, a qualitative function can be introduced to describe the correlation between local stress $\sigma_{loc}$ and *K*(*h*), that is, $\sigma_{loc}\;\propto \;K(h)$. Previous studies [41] have shown that, without UV, phenolic resin tends to debond at the fiber interface, while the fiber structure remains largely intact and difficult to remove effectively. This results in higher local fiber density and stress concentration, thereby increasing the risk of tearing and residual burrs. In contrast, the application of UV promotes uniform fracture of the phenolic resin and induces fiber breakage or shortening, leading to a looser local fiber and a reduced *K*(*h*) value. This redistribution helps reduce local stress concentration, facilitates compliant deformation of the material, improves fiber removal efficiency, and reduces the likelihood of burr formation.

### 2.2. Mesoscopic Scale: Deformation Features of Honeycomb Cells

The mesoscopic scale is between the microscopic scale and the macroscopic scale, and is commonly used to describe the response mechanism of a single structural unit under local stress or deformation behavior. As shown in Figure 1d, the deformation can be classified into three basic stress modes: horizontal, vertical, and shear [42]. Structurally, three adjacent honeycomb cells are connected by a common hinge point, and the three cell walls of each cell form the basic load-bearing unit around the hinge point. Therefore, local deformation of a single honeycomb cell can cause displacement of hinge points, thereby transmitting and inducing coordinated deformation of adjacent cells, ultimately leading to macroscopic structural collapse.

According to the elastic buckling analysis, when a honeycomb structure experiences local wall elastic instability under shear loading, the expression for its shear strength is:

(3) $$\tau_{L}^{*}=\frac{RE_{c}}{(1-v^{2})sin\alpha }(\frac{t}{s}{)}^{3}$$

(4) $$\tau_{W}^{*}=\frac{RE_{c}}{\left(1-v^{2})(1-cos\alpha \right)}(\frac{t}{s}{)}^{3}$$

where the honeycomb cell is a hexagonal structure with a thickness of *t*, a side length of *s*, and an angle of α between adjacent cell walls. $\tau_{L}^{*}$ is the shear strength of the honeycomb core in the *L* direction, $\tau_{W}^{*}$ is the shear strength of the honeycomb core in the *W* direction, *R* is a coefficient related to the constraint state, *E*_c_ is the elastic modulus of the honeycomb material, and *v* is Poisson’s ratio.

According to the plastic yielding analysis, when the loading continues to develop until the material reaches the yield strength, the expression for its shear strength is:

(5) $$\tau_{L}^{*}=\frac{\tau_{s}}{sin\alpha }\frac{t}{s}$$

(6) $$\tau_{W}^{*}=\frac{\tau_{s}}{1-cos\alpha }\frac{t}{s}$$

where $\tau_{s}$ is the yield strength of NHCs. In the above scenario, the relevant parameter values are as follows [43]: *s* = 1.83 mm, *t* = 0.06 mm, α = 120°, *R* = 2.5, and $\tau_{s}$ = 61 MPa.

Compared to the condition without UV, microscopic structural analysis indicates that UV reduces local fiber density and alleviates stress concentration, thereby facilitating more uniform deformation. This structural modulation can be qualitatively understood as a dynamic reduction in the equivalent elastic modulus *E*_c_, which, in turn, lowers the local resistance to shear and optimizes the failure mode.

### 2.3. Macroscopic Scale: Analysis of Cutting Mechanics

The macroscopic scale refers to the observable mechanical behavior of materials at the millimeter level or above. It includes force, deformation, and energy consumption. In the cutting process of NHCs, the interaction between the tool and the material exhibits obvious mechanical response characteristics, thus belonging to typical macroscopic scale behavior. As shown in Figure 1c, after the cutting edge of the tool cuts into the material, the honeycomb wall crack expands under the squeezing force *F*_n_ and friction force *F*_f_ of the cutting edge of the tool [11,44,45].

(7) $$\left\{\begin{array}{c} F_{x}=F_{n}\cos(arctan(\frac{V_{r}}{V_{f}}/\cos\theta )\times \left(\mu \cos\theta +\sin\theta \right)\\ F_{y}=F_{n}\sin(arctan(\frac{V_{r}}{V_{f}}/\cos\theta )\times \left(\mu \cos\theta +\sin\theta \right)\\ F_{z}=F_{n}\cos(arctan(\frac{V_{r}}{V_{f}}/\cos\theta )\times \left(\mu \cos\theta +\sin\theta \right) \end{array}\right.$$

where *μ* is the friction coefficient, *V*_r_ is the tangential velocity, *V*_f_ is the feed rate, and *θ* is the tool wedge angle. Microscopic structural features influence macroscopic mechanical parameters by affecting local mechanical responses. For example, as the *K*(*h*) value increases, the local fiber density becomes higher, leading to enhanced local stiffness and stress levels in the material. Thereby, this increases the resistance to tool penetration and significantly raises the normal squeezing force *F*_n_.

When the disc cutter is subjected to ultrasonic vibration, the cutting edge of the tool has an impact on the chips and the machined surface. Among them, the impact force of the rake face on the chips can accelerate the process of chip bending and separation. In addition, the tool and material are in intermittent contact [46,47,48], and the local cutting force of the circular cutting edge during ultrasonic cutting is:

(8) $$\left\{\begin{array}{l} F_{ux}={\frac{t_{s}}{T}F}_{n}\cos(arctan(\frac{V_{r}}{V_{f}}/\cos\theta )\times \left(\mu \cos\theta +\sin\theta \right)\\ F_{uy}=\frac{t_{s}}{T}F_{n}\sin(arctan(\frac{V_{r}}{V_{f}}/\cos\theta )\times \left(\mu +\sin\theta \right)\\ F_{uz}=\frac{t_{s}}{T}F_{n}\cos(arctan(\frac{V_{r}}{V_{f}}/\cos\theta )\times \left(\mu \sin\theta -\cos\theta \right)+F_{u}^{\prime } \end{array}\right.$$

where *t*_s_ is the effective time for the front cutting surface of the tool to come into contact with the chips within a single cycle, and $F_{u}^{\prime }$ is the total ultrasonic impact force of the tool on the chips.

By analyzing the theoretical model of cutting force, it can be concluded that the average cutting force *F*_ux_ within a single vibration cycle is significantly reduced compared to the cutting force without ultrasonic vibration. The cutting force *F*_ux_ decreases as the ratio of the tool tangential velocity *V*_r_ to the feed velocity *V*_f_ increases. In addition to the intermittent contact effect, ultrasonic vibration also contributes to the fiber distribution, resulting in a reduced *K*(*h*) value. This reduces local stiffness and stress concentration, further lowering the resistance to material removal. Therefore, ultrasonic vibration effectively alleviates tool load and facilitates more stable and efficient cutting by reducing the average cutting force.

## 3. Materials and Methods

### 3.1. Experimental Platform

**Figure 2:**
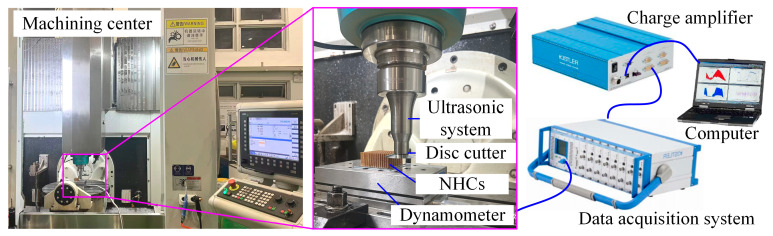
Experimental platform.

The experimental platform (Figure 2) was established on a five-axis machining center (EUMA DU810, Xuzhou, China) integrated with a specialized ultrasonic power system (UMINT-20-1000-Y, TsingDing, Shenzhen, China) designed for honeycomb composites processing. Cutting force data were acquired using a six-axis dynamometer (9119AA1, Kistler, Winterthur, Switzerland) with a sampling rate of 2 kHz. The dynamometer was connected to a charge amplifier (5080A100804, Kistler, Winterthur, Switzerland) and a data acquisition system (5697A, Kistler, Winterthur, Switzerland), ensuring accurate and real-time recording of force signals during the cutting process.

**Figure 3:**
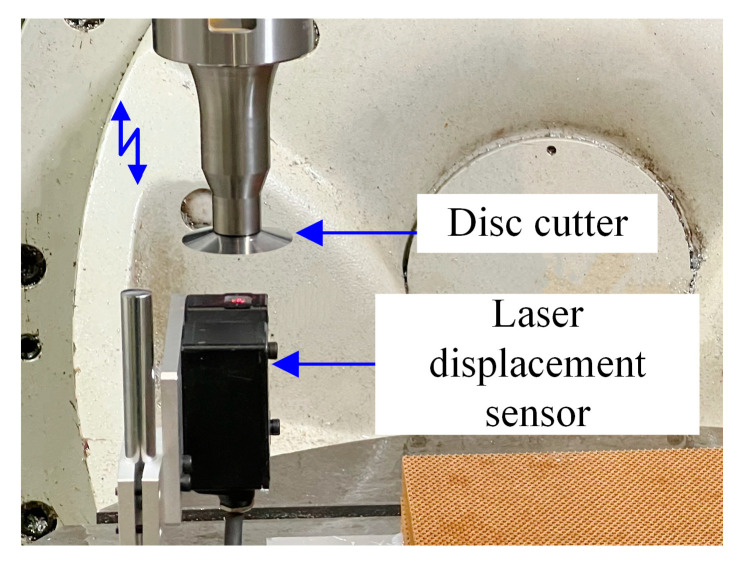
Schematic diagram of ultrasonic amplitude measurement.

The ultrasonic system was driven by a piezoelectric actuator and operated at a frequency of 20.22 kHz. The resulting tool-tip vibration amplitude reached 28 μm, which was precisely monitored using a high-resolution laser displacement sensor (LK-H025 series, Keyence, Osaka, Japan), as shown in Figure 3. This sensor, with a measurement accuracy of up to 1 μm, enabled accurate real-time tracking of the microscale vibration characteristics of the cutting tool during ultrasonic vibration-assisted machining.

**Figure 4:**
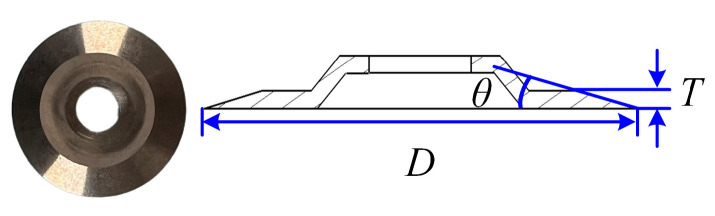
Schematic diagram of the disc cutter.

Table 1 NH-1-1.83-29, with an edge length *l* of 1.83 mm and a density *ρ* of 29 kg/m^3^. It belongs to the lightweight honeycomb material with low impregnation amount, and the detailed parameters are shown in Table 1. The disc cutter (Tungsten, Xiamen, China) is made of high-speed steel with good strength and toughness, and is easy to grind to form sharp cutting edges. The detailed parameters of the cutting tool are shown in Figure 4: the diameter *D* of the disc cutter is 25 mm, the wedge angle *θ* is set to 11°, and the thickness *T* is 0.8 mm.

### 3.2. Experimental Methods

To investigate the influence of UVA machining on the removal behavior of honeycomb composites, multi-scale cutting experiments were designed and conducted on NHCs. Orthogonal cutting tests were performed under both ultrasonic and non-ultrasonic conditions to compare differences in forces, structural deformation, and burrs. The relevant cutting parameters are detailed in Table 2. The experiment was repeated 5 times under each parameter condition to ensure the stability and reproducibility of the results. During the experiment, force sensors were used to record the mechanical responses. These responses were measured in the X, Y, and Z directions during the cutting process. The data was collected to obtain the dynamic trend of cutting force changes. In addition, the microstructure of the NHCs after processing was observed. An optical microscope was used for this observation. This allowed for the evaluation of the effect of material removal methods on surface integrity under different operating conditions. All experiments were conducted under the same basic conditions. The cutting depth was fixed at 1 mm. The cutting width was set to 10 mm. A constant vibration frequency of 20 kHz was maintained during ultrasonic vibration-assisted machining. This approach was used to eliminate interference from non-target variables on the experimental results.

## 4. Results and Discussion

### 4.1. Macroscopic Scale Results and Discussion

**Figure 5:**
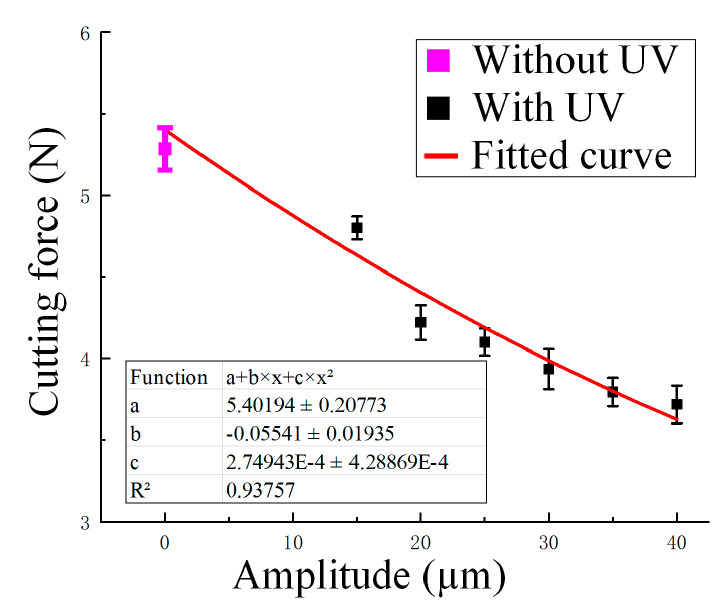
Cutting force (N) variation with ultrasonic amplitude (15–40 μm) under a constant feed rate of 3000 mm/min and spindle speed of 3000 r/min, with curve fitting applied. Pink: without UV; Black: with UV; Red line: fitted curve.

Based on experimental data, the cutting force in the macroscopic main feed direction showed variation trends under different ultrasonic amplitudes, feed rates, and spindle speeds. The effect of ultrasonic amplitude on cutting force is shown in Figure 5. The experimental results showed that the application of ultrasonic vibration effectively reduced the cutting force. Without UV, the average cutting force was 5.286 N. When UV was applied, the cutting force decreased progressively with increasing amplitude: 4.800 N at 15 μm, 4.220 N at 20 μm, 4.100 N at 25 μm, 3.934 N at 30 μm, 3.794 N at 35 μm, and 3.718 N at 40 μm. Compared to the condition without ultrasound, the cutting force at 40 μm amplitude was reduced by approximately 29.7%. This trend reflected the intermittent cutting mechanism introduced by UV, which periodically separated the tool from the material.

**Figure 6:**
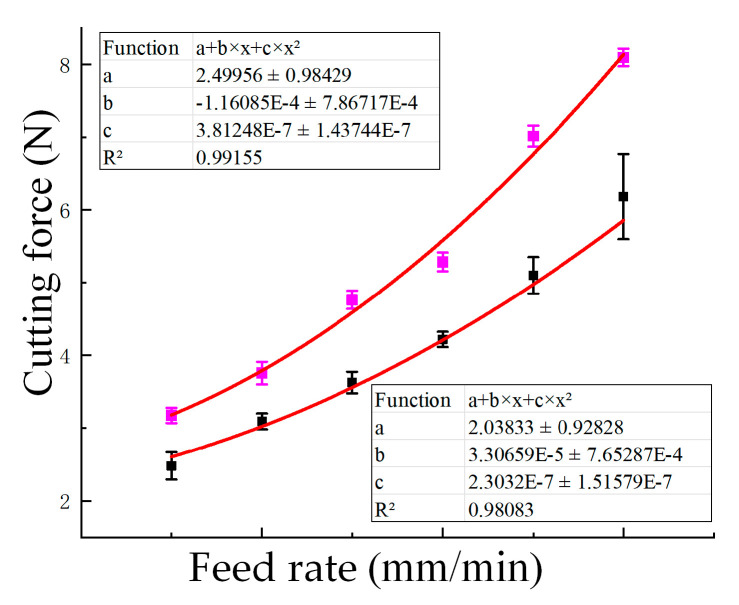
Cutting force (N) variation with feed rate (1500–4000 mm/min) under a constant ultrasonic amplitude of 20 μm and spindle speed of 3000 r/min, with curve fitting applied. Pink: without UV; Black: with UV; Red line: fitted curve.

The effect of feed rate on cutting force is shown in Figure 6, where both experimental results and fitted empirical curves are provided to reveal the underlying trend. The experimental results showed that the cutting force increased as the feed rate increased. Without UV, the cutting force rose from 3.176 N at 1500 mm/min to 8.096 N at 4000 mm/min, marking an increase of approximately 154.8%. Under UV, the cutting force increased from 2.482 N to 6.182 N over the same range, corresponding to a 149.1% increase. At each feed rate, ultrasonic assistance consistently resulted in a lower cutting force. For example, at 4000 mm/min, the reduction achieved by ultrasonic vibration was approximately 23.6%, indicating its effectiveness in lowering cutting resistance across different feed conditions.

**Figure 7:**
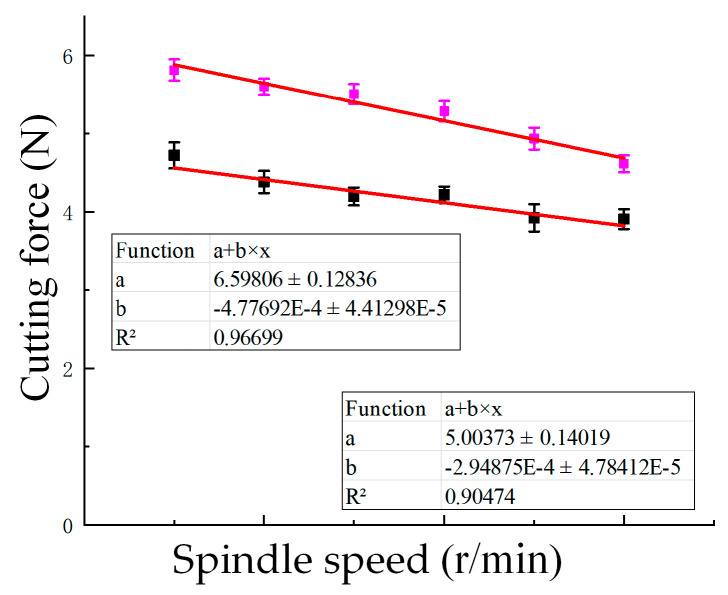
Cutting force (N) variation with spindle speed (1500–4000 r/min) under a constant ultrasonic amplitude of 20 μm and feed rate of 3000 mm/min, with curve fitting applied. Pink: without UV; Black: with UV; Red line: fitted curve.

The effect of spindle speed on cutting force is shown in Figure 7, and fitted empirical curves are provided to reveal the underlying trend. The increase in spindle speed had a relatively small impact on cutting force, but still showed a certain downward trend. Without UV, the average cutting force decreased from 5.814 N at 1500 r/min to 4.614 N at 4000 r/min, a reduction of approximately 20.6%. With UV, the cutting force dropped from 4.722 N to 3.910 N over the same spindle speed range, indicating a 17.2% reduction. At every tested spindle speed, ultrasonic machining produced lower cutting forces. For instance, at 1500 r/min, the cutting force was reduced by 18.8%, while at 4000 r/min, the reduction reached 15.2%, further confirming the beneficial effect of ultrasonic vibration in reducing cutting forces. In summary, ultrasonic vibration exhibits a good effect in reducing cutting force under different combinations of process parameters.

This article constructs a multi-parameter empirical analytical model based on measured cutting force data in the main feed direction. In contrast to previous cutting force prediction models [49], which overlook key influencing factors such as spindle speed, cutting width, and ultrasonic amplitude, making it difficult to accurately reflect the changes in cutting force under actual machining conditions. Therefore, this work proposes an optimized empirical formula for cutting force in the main feed direction, which comprehensively considers multiple process variables and their interactions. By considering these coupled effects, the proposed model demonstrates enhanced adaptability to real-world machining scenarios. As a result, it offers better generalization across different ultrasonic and machining conditions. It should be noted that the proposed model is developed under the following assumptions: the thermal effect during machining is minimal, boundary conditions are approximately uniform, and tool wear is negligible. The prediction equation is as follows:

(9) $$\begin{array}{c} F_{X}=\beta_{0}+\beta_{1}\cdot A+\beta_{2}\cdot B+\beta_{3}\cdot C+\beta_{4}\cdot D+\beta_{5}\cdot E+\beta_{6}\cdot F+\beta_{7}\cdot AC+\beta_{8}\cdot BC\\ +\beta_{9}\cdot B^{2}+\beta_{10}\cdot C^{2}+\beta_{11}\cdot DF+\beta_{12}\cdot F^{2} \end{array}$$

where *A* is the cutting depth (mm), *B* is the cutting width (mm), *C* is the feed rate (mm/min), *D* is the rotational speed (r/min), *E* is the tool radius (mm), and *F* is the amplitude (µm). Fit the curve based on the experimental values, as shown in Figure 5, Figure 6 and Figure 7, and then deduce and optimize the values of each parameter. In addition, the experimental data reveal that the cutting force is most strongly affected by the ultrasonic amplitude and feed rate, while spindle speed has a comparatively minor influence under the tested conditions.

(10) $$\begin{array}{c} F_{X}=1.53+0.03\cdot A-0.24\cdot B+4.68\times 10^{-4}\cdot C-2.12\times 10^{-5}\cdot D\\ \;\;\;\;\;\;\;\;\;\;\;\;\;\;\;+6.24\times 10^{-3}\cdot E-8.7\times 10^{-3}\cdot F+1.78\times 10^{-4}\cdot AC\\ \;\;\;\;\;\;\;\;\;\;\;\;\;\;\;-\;4.68\times 10^{-4}\cdot BC+3.32\times 10^{-2}\cdot B^{2}+2.05\times 10^{-7}\cdot C^{2}\\ -\;2.9\times 10^{-6}\cdot DF+4.01\times 10^{-5}\cdot F^{2} \end{array}$$

**Figure 8:**
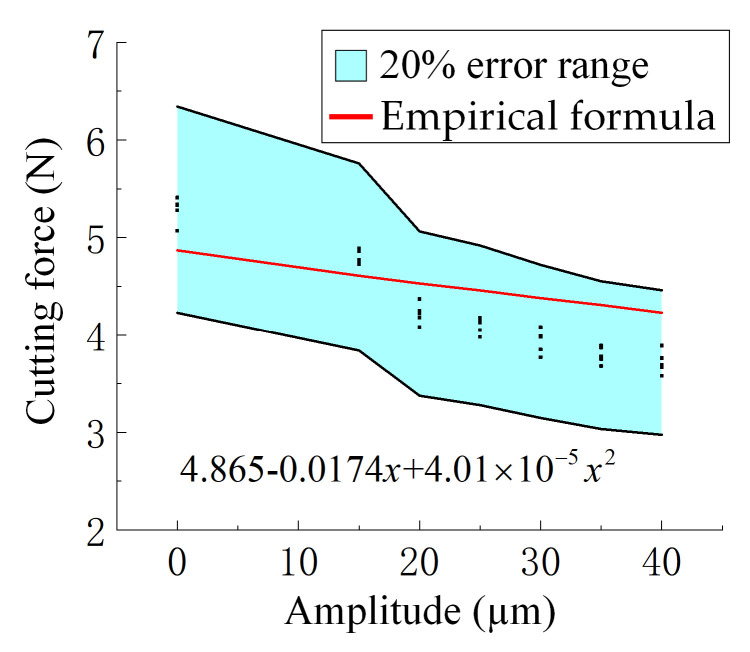
Cutting force versus ultrasonic amplitude with fitted curve based on Equation (11). The blue area indicates the ±20% error range, and the red line shows predicted values. Feed rate and spindle speed are fixed at 3000 mm/min and 3000 r/min, respectively.

**Figure 9:**
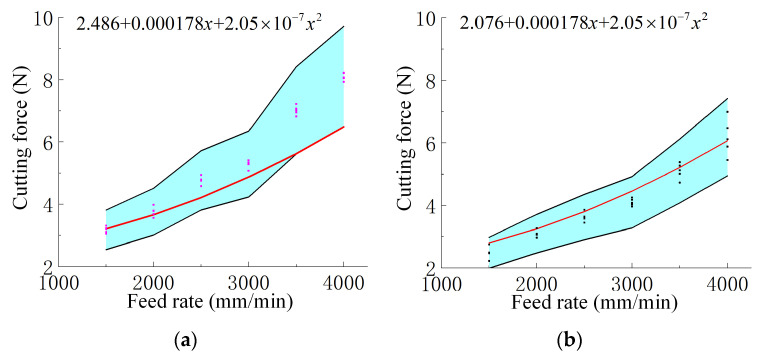
Cutting force versus feed rate and fitted curves based on Equation (11): (**a**) without UV and (**b**) with UV. Predictions fall within a ±20% error range. The blue area indicates the ±20% error range, and the red line shows predicted values. Ultrasonic amplitude and spindle speed are fixed at 20 μm and 3000 r/min, respectively.

**Figure 10:**
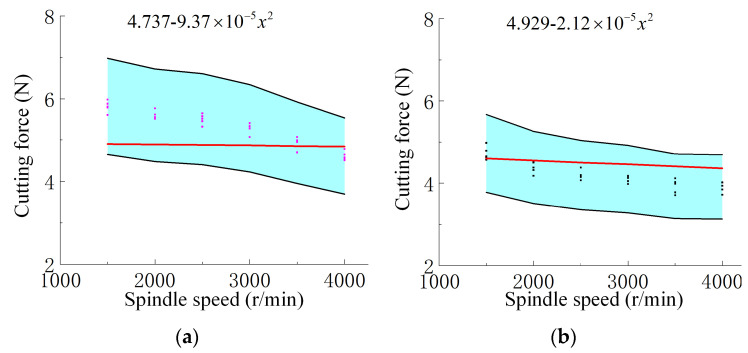
Cutting force versus spindle speed and fitted curves based on Equation (11): (**a**) without UV and (**b**) with UV. Predictions fall within a ±20% error range. The blue area indicates the ±20% error range, and the red line shows predicted values. Ultrasonic amplitude and feed rate are fixed at 20 μm and 3000 mm/min, respectively.

The blue-shaded region represents the ±20% error margin based on the averaged values from multiple sets of experimental measurements, while the scattered points denote the individual experimental data. The red curve is generated by substituting variables and constants into the newly derived empirical prediction formula (Equation (11)). It can be observed that all predictions fall within the acceptable error range, indicating good agreement between the model and experimental results. Figure 8 presents the empirical formula for cutting force with ultrasonic amplitude as the variable. Figure 9a,b show the cutting force prediction formulas under varying feed rates without and with ultrasonic vibration, respectively. Similarly, Figure 10a,b illustrate the empirical formulas for cutting force under different spindle speeds, in the absence and presence of ultrasonic vibration, respectively.

(11) $$\eta =\frac{\left|v-v_{p}\right|}{\left|v\right|}\times 100\%$$

where *η* is the relative error, *v* is the actual value observed, and $v_{p}$ is the prediction value. The average error between the predicted and measured values of the model is 8.6%, and the predicted curve is distributed within the error range of ±20% of the actual value, which verifies the accuracy and practicality of the model.

### 4.2. Mesoscopic Scale Results and Discussion

**Figure 11:**
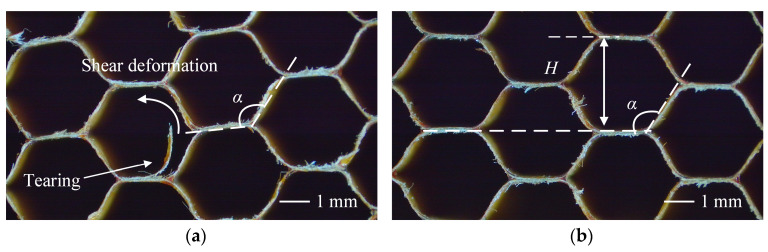
(**a**) Surface morphology without UV: feed rate and spindle speed at 3000 mm/min and 3000 r/min, respectively. (**b**) Surface morphology with UV: ultrasonic amplitude at 20 μm, spindle speed at 3000 r/min, and feed rate at 3000 mm/min.

The shear deformation of the honeycomb cell can be measured by the change in the angle α between adjacent honeycomb walls, which reflects the degree of damage to the microstructure (Figure 11). In the ideal undeformed state, the honeycomb cells are arranged in a regular hexagonal pattern with an angle of 120°. During the processing, external loads can cause compression and buckling of the edge walls of the cell, resulting in a deviation of the angle. The degree of deviation can be regarded as a characteristic indicator of the degree of local structural damage. Obtain the average cell angle under different processing parameters through experiments.

**Figure 12:**
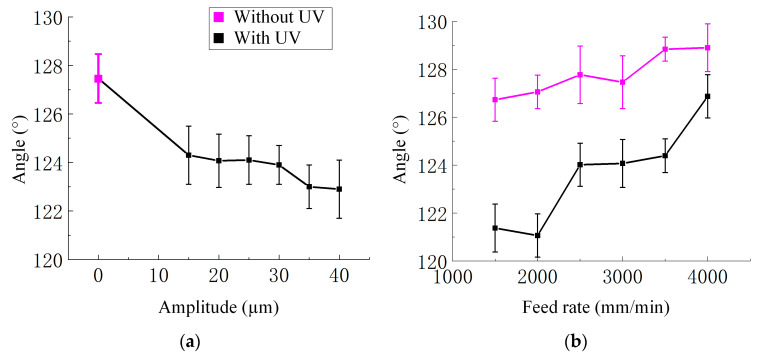
(**a**) The relationship curve between angle and ultrasound amplitude variation; experimental conditions: feed rate 3000 mm/min and spindle speed 3000 r/min. (**b**) The relationship curve between the angle and the feed rate with and without UV. Experimental conditions: ultrasonic amplitude 20 μm and spindle speed 3000 r/min.

Firstly, the angle decreased as the ultrasonic amplitude increased. Without UV, the angle was 127.46°. When UV was applied, the angle progressively decreased to 124.30° at 15 μm, 124.07° at 20 μm, 124.10° at 25 μm, 123.90° at 30 μm, 123.00° at 35 μm, and 122.90° at 40 μm. Compared to the non-ultrasonic condition, the angle was reduced by approximately 3.57% at the highest amplitude. This downward trend indicated that ultrasonic excitation altered the deformation behavior during cutting, resulting in a smaller deflection angle. The quantitative relationship between amplitude and angle is shown in Figure 12a.

Secondly, the effect of feed rate on angle is shown in Figure 12b. Under ultrasonic assistance (amplitude of 20 μm), the angle first slightly decreased and then gradually increased with increasing feed rate. Specifically, the angle decreased from 121.38° at 1500 mm/min to 121.07° at 2000 mm/min, then increased to 124.02°, 124.07°, 124.40°, and 126.87° at 2500, 3000, 3500, and 4000 mm/min, respectively. Overall, the trend was upward, with an approximate increase of 4.53% from the lowest to the highest feed rate. This behavior suggested a combined effect of material softening at lower feed rates and increased deformation resistance at higher feed rates. In the absence of ultrasonic vibration, the cutting angle consistently increased with rising feed rate, from 126.73° at 1500 mm/min to 128.90° at 4000 mm/min, reflecting an approximate increase of 1.71%. Intermediate values were 127.06°, 127.77°, 127.46°, and 128.84° at 2000, 2500, 3000, and 3500 mm/min, respectively. These results indicated that higher feed rates led to greater material resistance and larger deformation angles during cutting. Across all feed rates, the cutting angles under ultrasonic vibration were consistently smaller than those without vibration. For example, at 4000 mm/min, ultrasonic assistance reduced the angle by approximately 1.57%, confirming its mitigating effect on deformation.

Based on the comprehensive experimental data, the following damage area prediction model is proposed:

(12) $$S=S_{0}+K_{1}V_{f}+K_{2}A$$

where *S* is the predicted damage area (mm^2^), *S*_0_ is experimental measured damage area (mm^2^), *V*_f_ is the feed rate (mm/min), *A* is the ultrasonic amplitude (µm), and *K*_1_ and *K*_2_ are the fitting parameters, representing the strength of the influence of feed rate and amplitude on damage area changes, respectively. The damage area is defined as the area of a single honeycomb cell. A regular hexagon with an opposite side distance of *H* = 3.17 mm and internal angle *α* = 120° is taken as the reference, with an ideal area of approximately 8.70 mm^2^. To develop a predictive model for the damaged area under deformation, it is assumed that the honeycomb cell maintains a symmetric, equilateral hexagonal shape that undergoes compression, leading to changes in the internal angle *α* while keeping *H* constant. Although minor reductions in *H* were observed experimentally, the variation was small and, therefore, neglected for modeling purposes to improve clarity and computational efficiency. Under this assumption, the damage area can be estimated using the geometric formula:

(13) $$S_{0}=\frac{2}{3}H^{2}\cot\left(\frac{\alpha }{2}\right)$$

In the process of data fitting, when the dimensional system of each variable remains consistent, the dimensional conversion step can be omitted, and parameter estimation can be directly based on dimensionless values. The values are:

(14) $$S=7.95+0.00017V_{f}-0.012A$$

To evaluate the accuracy of this data-driven empirical equation, additional experiments were conducted under varied parameter settings. The predicted trends were compared with experimental outcomes, as summarized in Table 3.

Within the current experimental parameter range, a linear model was established to predict damage area as a function of feed rate and ultrasonic amplitude. Despite its simplicity, the prediction model effectively captures the primary trends in cell deformation behavior. The average error is 10.5%. With a compact form and limited parameters, it enables rapid evaluation of process effects while maintaining strong engineering applicability and computational efficiency. The relative error demonstrates that the model meets the accuracy requirements for both experimental analysis and practical application.

### 4.3. Microscopic Scale Results and Discussion

**Figure 13:**
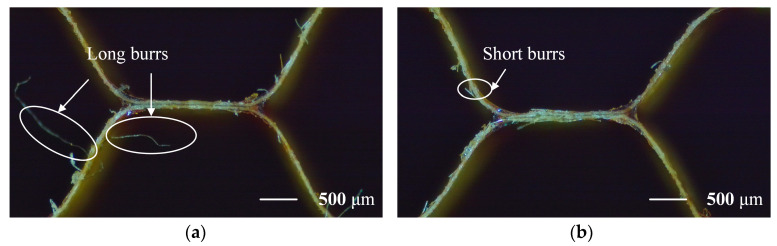
(**a**) Burr length without UV, feed rate, and spindle speed at 3000 mm/min and 3000 r/min, respectively. (**b**) Burr length with UV, ultrasonic amplitude at 20 μm, spindle speed at 3000 r/min, and feed rate at 3000 mm/min.

To reveal the regulatory mechanism of UVA machining on the formation behavior of micro burrs, the average maximum length of burrs appearing in the observation area under different feed rates and amplitudes were statistically analyzed (Figure 13). The experimental results indicate that the length of burrs is significantly affected by process parameters, especially exhibiting clear regularity under ultrasonic vibration assisted conditions.

**Figure 14:**
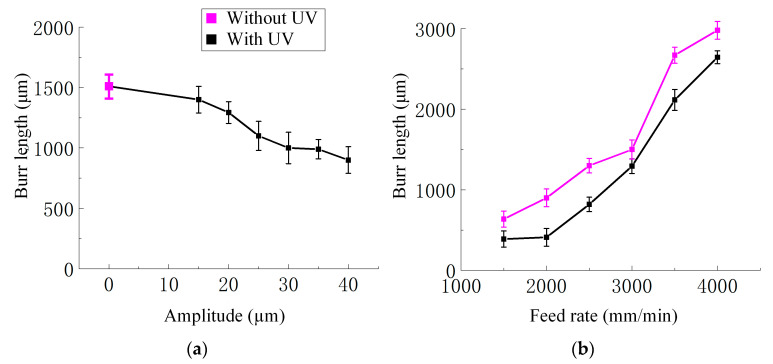
(**a**) The relationship curve between burr length and ultrasonic amplitude variation; experimental conditions: feed rate 3000 mm/min and spindle speed 3000 r/min. (**b**) The relationship curve between burr length and feed rate variation. Experimental conditions: ultrasonic amplitude 20 μm and spindle speed 3000 r/min.

Firstly, the quantitative relationship between amplitude and burr length is shown in Figure 14a. As the amplitude gradually increased from 0 μm to 40 μm, the maximum length of burrs gradually decreased from about 1510 μm to 900 μm, indicating that ultrasonic vibration effectively reduces local fiber warping and matrix tearing in the machining area by reducing the peak cutting force and material plastic deformation.

Secondly, the increase in feed rate significantly exacerbated the formation of burrs, as shown in Figure 14b. Without UV, as the feed rate gradually increased from 1500 mm/min to 4000 mm/min, the maximum length of burrs gradually increased from about 636 μm to 2980 μm. With UV, as the feed rate gradually increases from 1500 mm/min to 4000 mm/min, the maximum length of burrs gradually increases from about 389 μm to 2645 μm. Regardless of whether UV is applied, the length of burrs increases rapidly with the increase in feed rate. This is mainly due to the higher feed rate, which reduces the contact time between the tool and the material, and the shear zone cannot fully release the deformation energy, resulting in severe plastic deformation of the material at high strain rates, causing the fibers to be stretched and lifted instead of neatly cut, ultimately forming more significant burrs at the processing edge. In addition, under the same feed rate conditions, UV can effectively reduce burrs length. These trends are consistent with the observed decrease in macroscopic cutting force, further confirming the positive role of ultrasonic vibration. In contrast, the change in spindle speed has a relatively small impact on burr length, and it can be considered that it is not the main influencing factor within the parameter range of this experiment.

In this study, burr length is defined as the maximum extension distance from the honeycomb wall to the free end of the burr, measured along the main growth direction of the burr. The burr direction is defined as the direction perpendicular to the honeycomb wall, which serves as the reference orientation for burr formation. Based on comprehensive experimental data, the following empirical burr length prediction model is proposed:

(15) $$l=l_{0}+K_{1}V_{f}+K_{2}A$$

where *l* is the predicted burr length (µm), *l*_0_ is the reference length, *V*_f_ is the feed rate (mm/min), *A* is the ultrasonic amplitude (µm), and *K*_1_ and *K*_2_ are the fitting parameters, representing the strength of the influence of feed rate and amplitude on angle changes, respectively. In the process of data fitting, when the dimensional system of each variable remains consistent, the dimensional conversion step can be omitted, and parameter estimation can be directly based on dimensionless values. The values are:

(16) $$l=-964+0.946V_{f}-23.8A$$

To evaluate the accuracy of this data-driven empirical equation, additional experiments were conducted under varied parameter settings. The predicted trends were compared with experimental outcomes, as summarized in Table 4.

Within the current experimental parameter range, a linear model was established to predict burr length as a function of feed rate and ultrasonic amplitude. Despite its simplicity, the prediction model effectively captures the primary trends in burr formation behavior. The average error is 5.6%. With a compact form and limited parameters, it enables rapid evaluation of process effects while maintaining strong engineering applicability and computational efficiency. The relative error demonstrates that the model meets the accuracy requirements for both experimental analysis and practical application.

## 5. Conclusions

This study presents a comprehensive multi-scale investigation into the cutting behavior of Nomex honeycomb composites under ultrasonic vibration-assisted machining. The influence of UV on cutting force, structural deformation, and burr length was experimentally investigated, and the observed trends were captured by fitted empirical equations across multiple scales. It should be noted that the current empirical equations for burr length and deformation area are based on linear fit and do not account for potential interactions between processing parameters. This limitation may reduce prediction accuracy under complex coupling conditions. Future work could address this issue by introducing nonlinear terms or adopting data-driven approaches such as artificial neural networks to improve generalizability. Further validation in real-world industrial applications, such as the aeronautics industry, would enhance the applicability of these findings and support the development of optimized ultrasonic vibration-assisted machining strategies for a broader range of materials.

(1) At the macroscopic scale, the prediction error for the average cutting force in the feed direction is 8.6%. The application of ultrasonic vibration led to a reduction in cutting force across different amplitudes, feed rates, and spindle speeds. This reduction results from the intermittent contact mechanism introduced by the vibration, which lowers cutting resistance. Notably, the maximum reduction in cutting force reached approximately 29.7% at an amplitude of 40 μm.
(2) At the mesoscopic scale, the prediction error for the structural deformation analytical model for the damage area is less than 10.5%. The degree of deformation in honeycomb cells was assessed by analyzing the variation in cell angle α. Experimental results showed that ultrasonic vibration reduced the deformation from the regular hexagonal structure, reflecting a mitigating effect on structural damage.
(3) At the microscopic scale, the prediction error for the burr length at normal direction in orthogonal experiments is 5.6%. As the ultrasonic amplitude increased, the maximum burr length was reduced by up to 36.7%. Conversely, higher feed rates led to more long burrs due to insufficient material separation. Ultrasonic vibration was shown to reduce burr length by limiting fiber pull-out and matrix tearing.

## Figures and Tables

**Table 1:** Properties of NHCs.

Properties	Value
Type	NH-1-1.83-29
Density	29 kg/m^3^
Length	1.83 mm
Size	100 mm × 100 mm × 50 mm

**Table 2:** The parameters of cutting experiments with and without ultrasonic vibration.

Group	Ultrasonic Amplitude (μm)	Feed Rate (mm/min)	Spindle Speed (r/min)
1	15	3000	3000
2	20	3000	3000
3	25	3000	3000
4	30	3000	3000
5	35	3000	3000
6	40	3000	3000
7	20	1500	3000
8	20	2000	3000
9	20	2500	3000
10	20	3000	3000
11	20	3500	3000
12	20	4000	3000
13	20	3000	1500
14	20	3000	2000
15	20	3000	2500
16	20	3000	3000
17	20	3000	3500
18	20	3000	4000
19	0	1500	3000
20	0	2000	3000
21	0	2500	3000
22	0	3000	3000
23	0	3500	3000
24	0	4000	3000
25	0	3000	1500
26	0	3000	2000
27	0	3000	2500
28	0	3000	3000
29	0	3000	3500
30	0	3000	4000

**Table 3:** Empirical prediction results of the damage area based on experimentally fitted models.

Feed Rate (mm/min)	Amplitude (μm)	Experimental Value (mm^2^)	Predicted Value (mm^2^)	Relative Error
3000	40	8.14	7.98	2.3%
4000	20	7.64	8.39	9.8%
4000	0	7.22	8.63	19.5%

**Table 4:** Empirical prediction results of the burr length based on experimentally fitted models.

Feed Rate (mm/min)	Amplitude (μm)	Experimental Value (μm)	Predicted Value (μm)	Relative Error
3000	40	900.0	922.0	2.44%
4000	20	2645.3	2344.0	11.39%
4000	0	2980.2	2892.0	2.96%

## Data Availability

The data presented in this study are available from the corresponding authors upon a reasonable request.

## Abbreviations

The following abbreviations are used in this manuscript:

NHCs	Nomex honeycomb composites
DC	Disc cutter
UVA	Ultrasonic vibration-assisted
UV	Ultrasonic vibration

## References

[1] Yuan X., Zhang K., Zha H., Xu J., Song G., Cao W., Feng P., Feng F. (2023). Enabling Thin-Edged Part Machining of Nomex Honeycomb Composites via Optimizing Variable Angle of Disc Cutters. Materials.

[2] Liang Y., Feng P., Song Z., Zhu S., Wang T., Xu J., Yue Q., Jiang E., Ma Y., Song G. (2025). Wear Mechanisms of Straight Blade Tool by Dual-Periodic Impact Platform. Int. J. Mech. Sci..

[3] Singh R.P., Singhal S. (2016). Rotary Ultrasonic Machining: A Review. Mater. Manuf. Process..

[4] Thoe T., Aspinwall D., Wise M. (1998). Review on Ultrasonic Machining. Int. J. Mach. Tools Manuf..

[5] Ahmad S., Zhang J., Feng P., Yu D., Wu Z., Ke M. (2020). Processing Technologies for Nomex Honeycomb Composites (NHCs): A Critical Review. Compos. Struct..

[6] Guo J., Sun J., Du H., Zhang Y., Dong Z., Kang R., Wang Y. (2024). Cutting Force Reduction Mechanism in Ultrasonic Cutting of Aramid Honeycomb. Int. J. Mech. Sci..

[7] He M., Hu W. (2008). A Study on Composite Honeycomb Sandwich Panel Structure. Mater. Des..

[8] Grove S.M., Popham E., Miles M.E. (2006). An Investigation of the Skin/Core Bond in Honeycomb Sandwich Structures Using Statistical Experimentation Techniques. Compos. Part A Appl. Sci. Manuf..

[9] Galletti G.G., Vinquist C., Es-Said O.S. (2008). Theoretical Design and Analysis of a Honeycomb Panel Sandwich Structure Loaded in Pure Bending. Eng. Fail. Anal..

[10] Yuan X., Li B., Feng F., Xu J., Song G., Liang Y., Ma Y., Xu C., Wang F., Feng P. (2024). False Boss Connection for Precision Machining of Composites with Soft and Brittle Characteristics. J. Compos. Sci..

[11] Xiang D., Wu B., Yao Y., Zhao B., Tang J. (2019). Ultrasonic Vibration Assisted Cutting of Nomex Honeycomb Core Materials. Int. J. Precis. Eng. Manuf..

[12] Tian J., Bao Y., Chen K., Dong Z., Kang R., Guo D., Sun J. (2025). Prediction and Analysis of Grinding Burr of CFRP Circular Tube with a Rounded Corner Grinding Wheel. J. Manuf. Process..

[13] Sun J., Qin Y., Xing W., Kang R., Dong Z., Wang Y. (2024). Study on Cell Wall Deformation in Ultrasonic Cutting Aluminum Honeycomb by Straight-Blade Knife. Ultrasonics.

[14] Wang Z., Li Z., Zhou W., Hui D. (2018). On the Influence of Structural Defects for Honeycomb Structure. Compos. Part B.

[15] Sun J., Wang Y., Zhou P., Wang M., Kang R., Dong Z. (2023). Equivalent Mechanical Model of Resin-Coated Aramid Paper of Nomex Honeycomb. Int. J. Mech. Sci..

[16] Qin Y., Kang R., Wang Y., Sun J., Dong Z. (2022). A Form Error Evaluation Method of Honeycomb Core Surface. Measurement.

[17] Xu J., Feng P., Gong Y., Wang J., Yang H., Feng F. (2025). Exploiting Damage for Inhibiting Damage: A Counterintuitive Reasoning out of in-Situ Orthogonal Cutting for Brittle Fiber Composite. J. Mater. Process. Technol..

[18] Kang J., Zhang H., Zhang Z., Bai T., Zuo C., Guo J., Zhang J. (2024). Investigating Damage Mechanisms of Honeycomb Cores Machined with PCD Circular Saw Blades. J. Mater. Process. Technol..

[19] Jiang E., Yue Q., Xu J., Fan C., Song G., Yuan X., Ma Y., Yu X., Yang P., Feng P. (2024). A Wear Testing Method of Straight Blade Tools for Nomex Honeycomb Composites Machining. Wear.

[20] Xu J., Zhang K., Zha H., Liu J., Yuan X., Cai X., Xu C., Ma Y., Feng P., Feng F. (2023). Surface Integrity of Nomex Honeycomb Composites after Ultrasonic Vibration Machining by Using Disc Cutters. J. Manuf. Process..

[21] Xu J., Yue Q., Zha H., Yuan X., Cai X., Xu C., Ma Y., Feng P., Feng F. (2023). Wear Reduction by Toughness Enhancement of Disc Tool in Nomex Honeycomb Composites Machining. Tribol. Int..

[22] Lan T., Feng P., Zhang J., Zhou H., Wang J. (2023). Modeling the Load Capacity of Frequency-Tracked Rotary Ultrasonic Machining System. Int. J. Mech. Sci..

[23] Wang Y., Kang R., Dong Z., Wang X., Huo D., Zhang X. (2021). A Novel Method of Blade-Inclined Ultrasonic Cutting Nomex Honeycomb Core with Straight Blade. J. Manuf. Sci. Eng..

[24] McCarthy C.T., Annaidh A.N., Gilchrist M.D. (2010). On the Sharpness of Straight Edge Blades in Cutting Soft Solids: Part II—Analysis of Blade Geometry. Eng. Fract. Mech..

[25] Huang X. (2015). Research on Ultrasonic Cutting Mechanism of Nomex Honeycomb Composites Based on Fracture Mechanics. J. Mech. Eng..

[26] Shan S., Feng P., Zha H., Feng F. (2020). Building of Longitudinal Ultrasonic Assisted Turning System and Its Cutting Simulation Study on Bulk Metallic Glass. Materials.

[27] Jiang J., Liu Z. (2021). Formation Mechanism of Tearing Defects in Machining Nomex Honeycomb Core. Int. J. Adv. Manuf. Technol..

[28] Xiang D., Wu B., Yao Y., Liu Z., Feng H. (2019). Ultrasonic Longitudinal-Torsional Vibration-Assisted Cutting of Nomex^®^ Honeycomb-Core Composites. Int. J. Adv. Manuf. Technol..

[29] Wang Y., Kang R., Qin Y., Meng Q., Dong Z. (2021). Effects of Inclination Angles of Disc Cutter on Machining Quality of Nomex Honeycomb Core in Ultrasonic Cutting. Front. Mech. Eng..

[30] Zarrouk T., Nouari M., Salhi J.-E., Benbouaza A. (2024). Numerical Simulation of Rotary Ultrasonic Machining of the Nomex Honeycomb Composite Structure. Machines.

[31] Zarrouk T., Salhi J.-E., Nouari M., Bouali A. (2024). Enhancing the Machining Performance of Nomex Honeycomb Composites Using Rotary Ultrasonic Machining: A Finite Element Analysis Approach. Materials.

[32] Zarrouk T., Nouari M., Salhi J.-E. (2024). Numerical Study on Rotary Ultrasonic Machining (RUM) Characteristics of Nomex Honeycomb Composites (NHCs) by UCSB Cutting Tool. Int. J. Adv. Manuf. Technol..

[33] Zarrouk T., Salhi J.-E., Nouari M., Barboucha M. (2025). Modeling of Nomex Honeycomb Structure Milling Assisted by Longitudinal–Torsional Vibrations with a CZ10 Combined Tool: Optimization of Tool Wear and Surface Integrity. Appl. Mech..

[34] Ahmad S., Zhang J., Feng P., Yu D., Wu Z. (2020). Experimental Study on Rotary Ultrasonic Machining (RUM) Characteristics of Nomex Honeycomb Composites (NHCs) by Circular Knife Cutting Tools. J. Manuf. Process..

[35] Xu J., Wang C., Feng P., Jiang E., Feng F. (2023). Meso-Scale Cracks Initiation of Nomex Honeycomb Composites in Orthogonal Cutting with a Straight Blade Cutter. Compos. Sci. Technol..

[36] Roy R., Nguyen K.H., Park Y.B., Kweon J.H., Choi J.H. (2014). Testing and Modeling of Nomex^TM^ Honeycomb Sandwich Panels with Bolt Insert. Compos. Part B Eng..

[37] Qin Y., Kang R., Dong Z., Wang Y., Yang J., Zhu X. (2018). Burr Removal from Measurement Data of Honeycomb Core Surface Based on Dimensionality Reduction and Regression Analysis. Meas. Sci. Technol..

[38] Dong Z., Qin Y., Kang R., Wang Y., Sun J., Zhu X., Liu Y. (2021). Robust Cell Wall Recognition of Laser Measured Honeycomb Cores Based on Corner Type Identification. Opt. Lasers Eng..

[39] Wang W., Dai Y., Zhang C., Gao X., Zhao M. (2016). Micromechanical Modeling of Fiber-Reinforced Composites with Statistically Equivalent Random Fiber Distribution. Materials.

[40] Ramsden J.J. (1993). Review of New Experimental Techniques for Investigating Random Sequential Adsorption. J. Stat. Phys..

[41] Sun D., Kang R., Wang Y., Guo J., Dong Z. (2020). A Novel Ultrasonic Trepanning Method for Nomex Honeycomb Core. Appl. Sci..

[42] Bianchi G., Aglietti G.S., Richardson G. (2012). Static and Fatigue Behaviour of Hexagonal Honeycomb Cores under In-Plane Shear Loads. Appl. Compos. Mater..

[43] Jaafar M., Nouari M., Makich H., Moufki A. (2021). 3D Numerical Modeling and Experimental Validation of Machining Nomex^®^ Honeycomb Materials. Int. J. Adv. Manuf. Technol..

[44] Wang Y. (2017). Analysis of Influence on Ultrasonic-Assisted Cutting Force of Nomex Honeycomb Core Material with Straight Knife. J. Mech. Eng..

[45] Yang Y., Bao Y., Wang J., Chen C. (2022). Study on the Cutting Damage Mechanism of Aramid Honeycomb Based on the Progressive Damage Model. Materials.

[46] Yang Z., Zhu L., Zhang G., Ni C., Lin B. (2020). Review of Ultrasonic Vibration-Assisted Machining in Advanced Materials. Int. J. Mach. Tools Manuf..

[47] Nath C., Rahman M. (2008). Effect of Machining Parameters in Ultrasonic Vibration Cutting. Int. J. Mach. Tools Manuf..

[48] Ni C., Zhu L., Liu C., Yang Z. (2018). Analytical Modeling of Tool-Workpiece Contact Rate and Experimental Study in Ultrasonic Vibration-Assisted Milling of Ti–6Al–4V. Int. J. Mech. Sci..

[49] Cao W., Zha J., Chen Y. (2020). Cutting Force Prediction and Experiment Verification of Paper Honeycomb Materials by Ultrasonic Vibration-Assisted Machining. Appl. Sci..

